# A 110–170 GHz Wideband LNA Design Using the InP Technology for Terahertz Communication Applications

**DOI:** 10.3390/mi14101921

**Published:** 2023-10-10

**Authors:** Lian Hu, Ziqiang Yang, Yuan Fang, Qingfeng Li, Yixuan Miao, Xiaofeng Lu, Xuechun Sun, Yaxin Zhang

**Affiliations:** 1 School of Electronic Science and Engineering (National Exemplary School of Microelectronics), University of Electronic Science and Technology of China, Chengdu 610054, China; icui4cuhl@163.com (L.H.); njuptlqf@163.com (Q.L.); 2019050310008@std.uestc.edu.cn (Y.M.); lu_xfp01@163.com (X.L.); qiumimiz@163.com (X.S.); zhangyaxin@uestc.edu.cn (Y.Z.); 2 Huzhou Key Laboratory of Terahertz Integrated Circuits and Systems, Yangtze Delta Region Institute (Huzhou), University of Electronic Science and Technology of China, Huzhou 313001, China; 3 The 13th Research Institute, CETC, Shijiazhuang 050051, China; nicefangyuan@163.com

**Keywords:** high gain, wideband, LNA, terahertz, InP HEMT

## Abstract

This paper proposes a low-noise amplifier (LNA) for terahertz communication systems. The amplifier is designed based on 90 nm InP high-electron-mobility transistor (HEMT) technology. In order to achieve high gain of LNA, the proposed amplifier adopts a five-stage amplification structure. At the same time, the use of staggered tuning technology has achieved a large bandwidth of terahertz low-noise amplification. In addition, capacitors are used for interstage isolation, sector lines are used for RF bypass, and Microstrip is used to design matching circuits. The entire LNA circuit was validated using accurate electromagnetic simulation. The simulation results show that at 140 GHz, the small signal gain is 25 dB, the noise figure is 4.4 dB, the input 1 dB compression point is −19 dBm, and the 3 dB bandwidth reaches 60 GHz (110–170 GHz), which validates the effectiveness of the design.

## 1. Introduction

Technically, terahertz waves are electromagnetic waves with frequencies ranging from 100 GHz to 10 THz. Positioned between millimeter waves and visible light frequencies, the terahertz range partially overlaps with both infrared and millimeter waves. The unique location of terahertz waves has garnered significant interest among researchers, leading to extensive investigations into terahertz technology. Compared to light waves and millimeter waves, terahertz waves possess advantageous properties [1,2]. They exhibit better penetrability than light waves and offer more available bandwidth than millimeter waves. These distinctive characteristics have attracted scholars to delve deeper into the potential applications and capabilities of terahertz technology.

The Terahertz Low-Noise Amplifier (LNA) chip is a critical component of the terahertz system, situated in the first stage of the terahertz receiver. Its noise figure plays a pivotal role in determining the overall noise performance of the receiver. However, achieving a low-noise design in the terahertz frequency band poses considerable challenges due to the amplified active device noise and losses in matching circuits [3,4,5,6,7,8]. Moreover, as the frequency increases within the terahertz band, the maximum gain of the device tends to decrease significantly, which directly impacts the amplifier’s overall gain. Consequently, designing circuits that achieve both low-noise and high-gain amplification in the terahertz frequency band presents a formidable challenge.

To meet the demands for low noise and high gain at high frequencies, Indium-Phosphide (InP)-based High-Electron-Mobility Transistors (HEMTs) are preferred due to their excellent electromigration properties. The composite structure of InGaAs/InAlAs/InP with a gate length of 70 nm can further enhance the effective quality and elevate the effective Electron mobility and speed by increasing the indium content in the In(x)Ga(1 − x)As channel. This particular device achieves an impressive peak transconductance of 1600 mS/mm, with a maximum oscillation frequency reaching 310 GHz. Such capabilities make this InP transistor highly suitable for integration into terahertz circuits, offering great promise for applications in the terahertz frequency range [9,10].

In the LNA design proposed in this article, a technique is employed to reduce the noise introduced by the first stage. This is achieved by removing the bias resistance of the first stage and supplying it separately. Recognizing that the size and bias voltage of the transistor significantly impact power consumption and noise performance parameters, this study optimizes circuit performance by carefully selecting appropriate transistor sizes and bias voltages. Additionally, the paper employs interleaving tuning technology to achieve a substantial bandwidth. Through these efforts, the article ultimately presents an LNA circuit that boasts low noise, high gain, and wide bandwidth.

## 2. Technology and Transistor Sizing

**Figure 1:**
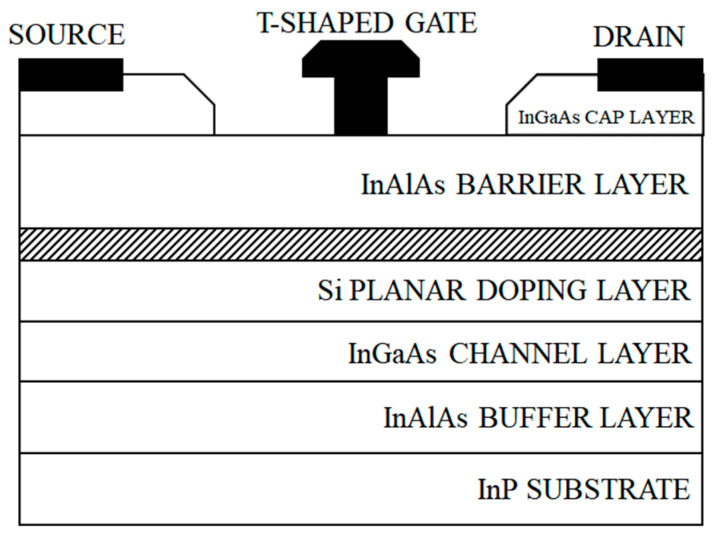
Schematic diagram of InP HEMT section structure.

InP devices are widely favored for their low-noise characteristics at high frequencies. To achieve optimal gain and noise figures in high-frequency, low-noise amplifiers, the physical structure design of InP HEMTs plays a crucial role. The construction of InP HEMT devices consists of several layers: the bottom layer being an InP substrate, with a buffer layer (InAlAs) positioned above it, and a channel layer (InGaAs) above the buffer layer. An InAlAs material is used as a spatial isolation layer above the channel, and the doped barrier layer is composed of InAlAs material. This is followed by the Schottky contact on the barrier layer and the top InGaAs cap layer. The structural schematic is illustrated in Figure 1.

**Figure 2:**
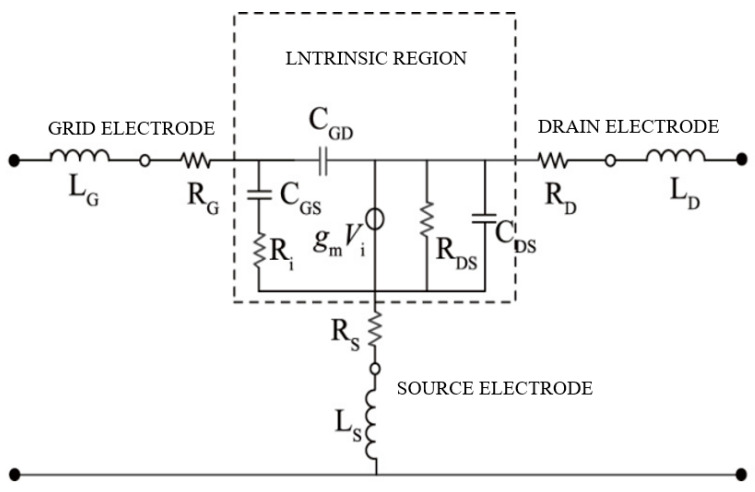
The small-signal equivalent circuit model of the InP HEMT device.

The prerequisite for designing a low-noise amplifier for terahertz InP HEMT lies in establishing the small-signal equivalent circuit model and noise equivalent circuit model of the InP HEMT device. The accurate establishment of the small-signal equivalent circuit model is of paramount importance as it forms the basis for constructing the noise equivalent circuit model. The extraction of parameters for the small-signal equivalent circuit model of the InP HEMT device includes parasitic capacitance extraction, parasitic inductance extraction, parasitic resistance extraction, and intrinsic component extraction. Based on the extracted parameters, the small-signal equivalent circuit model of the InP HEMT device, as depicted in Figure 2, was established, with the dashed box representing the intrinsic region of the equivalent circuit. Thoughtful consideration and precise establishment of these models are crucial to ensuring reliable and efficient circuit design.

After extracting data from various parameter measurements, a fitting process was conducted. Under the bias conditions of V_ds_ = 1.8 V and V_gs_ = 0 V, the small-signal model parameters were fitted and obtained as shown in Table 1. These parameters can be utilized for the investigation of the noise equivalent circuit model.

It is worth noting that the semiconductor device noise equivalent model is established based on the foundation of small-signal equivalent circuit models. In this design, the PRC model [11,12,13,14] is employed to simulate the noise characteristics of the device. The PCR model is a noise modeling methodology that finds particular suitability in noise analysis for transistors and various microelectronic devices. The acronym, PCR, stands for “Piecewise-Constant Recursive”, signifying that the model dissects noise sources into a sequence of discrete constant segments and subsequently calculates the overall noise through recursive methods. Usually, two noise current sources, $\bar{i_{g}^{2}}$ and $\bar{i_{d}^{2}}$, are used for equivalence. Its related noise can be expressed as

(1) $$\bar{i_{g}^{2}}=\frac{4kT∆f\omega^{2}C_{gs}^{2}R}{g_{m}}$$

(2) $$\bar{i_{d}^{2}}=4kT∆fg_{m}P$$

(3) $$\bar{i_{g}}\bar{i_{d}}=C\sqrt{\bar{i_{g}^{2}}\bar{i_{d}^{2}}}=4kT∆f\omega^{2}C_{gs}^{2}C\sqrt{PR}$$

In the formula, C is the relevant noise factor; k is the Boltzmann constant; T is the temperature; ∆f is the frequency variation; ω Is the angular frequency; $C_{gs}$ is the gate source capacitor; P is the noise factor of the drain channel; R is the gate induced noise factor. In the design of low noise amplification circuits, accurately characterizing the noise parameters is of utmost importance to achieve optimal noise impedance and achieve low noise matching. By taking into account the various noise sources, the design aims to minimize noise interference and enhance the overall circuit performance, especially in applications where low noise is critical. This approach ensures the circuit operates with reduced noise levels, making it well-suited for sensitive applications that require high signal-to-noise ratios and reliable performance.

**Table 1:** Small-signal parameters of 2 × 15 InP HEMT [15].

$L_{g}/pH$	$L_{d}/pH$	$L_{s}/pH$	$C_{pgs}/fF$	$C_{pgd}/fF$	$R_{s}/\Omega$
52	62.3	5.81	47.2	34.7	61.3
$R_{d}/\Omega$	$R_{g}/\Omega$	$C_{ds}/fF$	$C_{gs}/fF$	$C_{gd}/fF$	$R_{ds}/\Omega$
14.2	32.2	300	296	11.4	296.3
$R_{gs}/\Omega$	$g_{m}/S$	τ/ps			
50	150	17			

**Figure 3:**
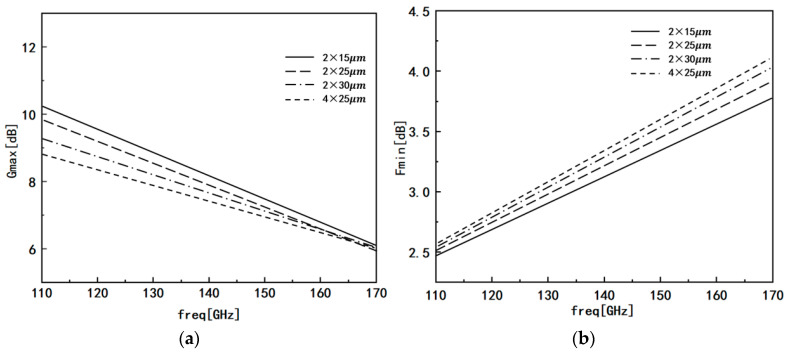
Performance comparison of devices of different sizes: (**a**) maximum available gain curve; (**b**) minimum noise figure curve.

The design specifications for this low-noise amplifier demand a gain greater than 25 dB, noise figure below 5 dB, and an input-to-output standing wave ratio of less than 3:1 within the operating frequency band of 110–170 GHz. To achieve optimal device size for desired performance, the design carefully considers the parasitic effects surrounding the transistors. By comparing devices with different gate widths and analyzing their maximum available gain (G_max_) and minimum noise figure (F_min_) curves, as shown in Figure 3, a comprehensive evaluation is performed.

Upon examining the curves, it becomes evident that at 140 GHz, the maximum available gain and minimum noise figure exhibit distinct trends for different device sizes. After rigorous comparison and analysis, the size that offered the best compromise between maximum available gain and minimum noise figure was selected. Eventually, a 2 × 15 μm low-noise device was chosen for the final design, effectively meeting the stringent performance requirements for the specified frequency range.

According to the amplifier design specifications, since the first stage is primarily focused on minimizing noise, it has a limited capacity to provide gain. Assuming that the subsequent gain stages can each achieve approximately 7 dB of gain, ideally, the combined gain for four stages would be around 25 dB. However, taking into consideration factors such as losses in transmission lines and the bandwidth limitations of matching structures, the overall gain will experience significant reductions. To ensure the desired final performance with a certain margin, this circuit will adopt a cascaded structure comprising five stages. The total noise figure (*F*) will be

(4) $$F=F_{1}+\frac{F_{2}-1}{G_{1}}+\frac{F_{3}-1}{G_{1}G_{2}}+\ldots +\frac{F_{5}-1}{G_{1}G_{2}G_{3}G_{4}G_{5}}$$

In the formula, $F_{1}$, $F_{2}$, …, $F_{5}$ and $G_{1}$, $G_{2}$, …, $G_{5}$ are the noise coefficients and gains of each stage, respectively. From the equation, it becomes evident that the noise performance of the first-stage amplifier significantly impacts the overall noise performance of the entire amplifier [16,17,18]. As a result, careful consideration must be given to optimizing the noise performance while selecting the operating point and designing the matching circuit. This ensures that the noise figure of the entire circuit meets the desired performance requirements.

**Figure 4:**
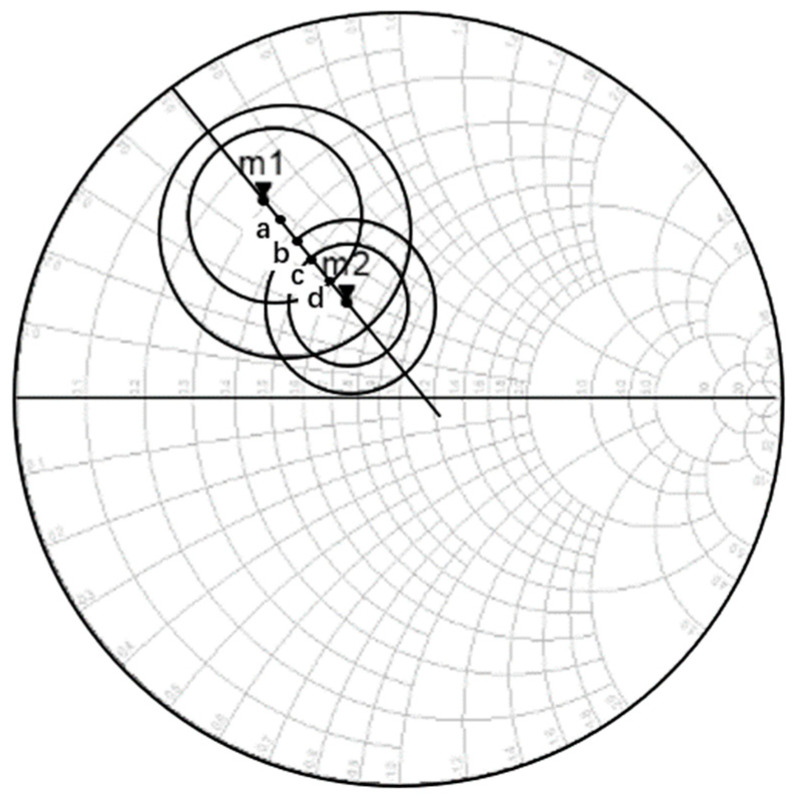
Equal gain, noise figure impedance circular diagram.

For the matching structure design of amplifiers, various factors such as stability, noise figure, gain, and standing wave need to be carefully considered. One of the most critical aspects of the design is the selection of impedance points. Figure 4 illustrates the impedance circle with equal gain and noise coefficient. In the figure, point m1 represents the impedance point with maximum available gain, while point m2 represents the impedance point with the minimum noise figure. Points a, b, c, and d represent four impedance points evenly distributed along the line connecting m1 and m2. When selecting the impedance point for the first stage amplifier, the noise performance should be taken into account. However, designing the amplifier solely based on the minimum noise requirements may result in a large mismatch of the source impedance, leading to a decline in power gain and deterioration in the input standing wave ratio. To address this, it is essential to choose several impedance points at appropriate locations for matching design, aiming to strike a balance between noise coefficient and input standing wave ratio. Ultimately, the first stage amplifier design will be carried out by selecting point d. For the subsequent stages of the design, where achieving high-gain output is crucial, point m1 is directly chosen for maximum gain matching design. By thoughtfully considering these impedance points, the amplifier can be effectively optimized to achieve the desired performance in terms of gain, noise figure, and standing wave ratio.

**Figure 5:**
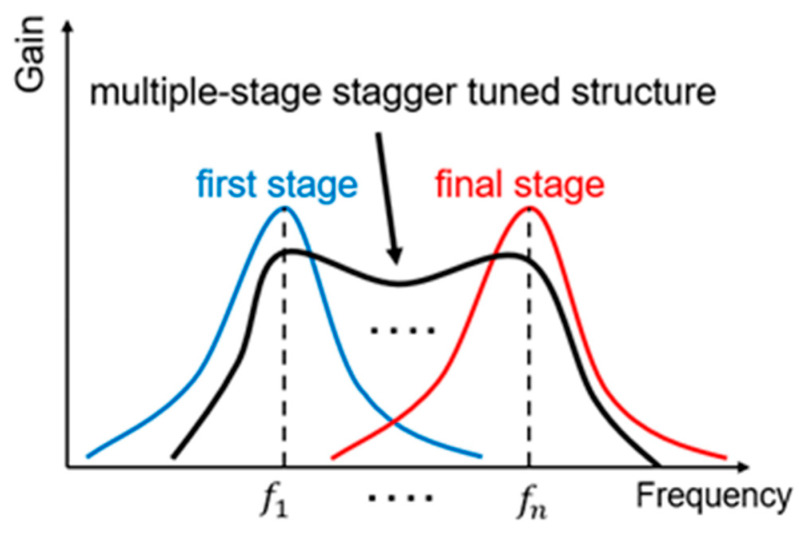
The effect of staggered tuning structure to broadband operation.

According to the frequency response characteristics of the matching network, the amplifier’s matching circuit may not always achieve the minimum noise figure or maximum gain matching across the entire frequency band. To achieve broadband operation, a staggered tuning approach is adopted, wherein the maximum gain frequency points of each stage are intentionally staggered [19,20]. Figure 5 illustrates the impact of the staggered tuning structure on broadband operation. By strategically staggering the tuning frequencies of each stage, the amplifier can effectively cover a wider frequency range while maintaining favorable gain and noise figure characteristics across the band. This approach allows for better performance over a broader spectrum, making the amplifier suitable for applications requiring broad frequency coverage and consistent performance.

Since the matching point for the first stage was chosen at the previously mentioned point “d,” the first stage represents a trade-off between gain and noise. Therefore, the target noise figure is around 3.6 dB, and the gain is approximately 4 dB. For the final four stages of the amplification circuit, a high-gain matching approach was employed, and the design was based on point “m1” as shown in Figure 4. The expected noise level for each stage is around 5.5 dB, while achieving a combined gain output of 28 dB. By incorporating the gain and noise of circuits at all levels into formula (1), the calculated noise figure of the amplifier at the center frequency of 140 GHz is approximately 4.2 dB, with a gain of around 32 dB. The design strives to strike a balance between noise and gain performance, optimizing the amplifier for efficient operation at the target frequency. By carefully managing the noise figure and gain at different stages, the amplifier is anticipated to deliver reliable and high-performance results in its operational range.

## 3. LNA Circuit Design

**Figure 6:**
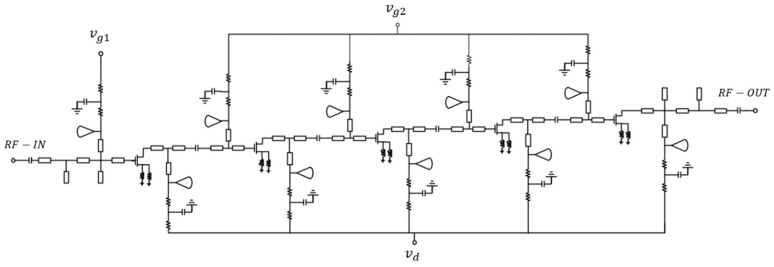
The schematic of the proposed wideband LNA.

The matching circuit of a low-noise amplifier serves multiple objectives, aiming not only to achieve low noise and high gain but also to improve the circuit’s stability and achieve appropriate power and standing wave ratio. For multi-stage cascaded amplifiers, the design of the matching network is divided into three parts: input matching, interstage matching, and output matching. Input matching focuses on achieving the best noise matching, while interstage matching aims for maximum gain matching. Output circuit matching is designed to reach 50 ohms. Due to the high frequency of the amplifier, high and low impedance lines are used to achieve impedance matching. Utilizing high and low impedance lines for matching can help optimize the amplifier’s performance, increase the bandwidth, reduce noise, and enhance stability. Moreover, for each stage of the amplifier circuit, radial stub lines, parallel capacitors, and resistive capacitor structures are employed for source and drain biases. These components help block bypass RF signals and improve the stability of the amplifier while achieving the required matching. The topology of the circuit is depicted in Figure 6.

## 4. Result and Discussion

**Figure 7:**
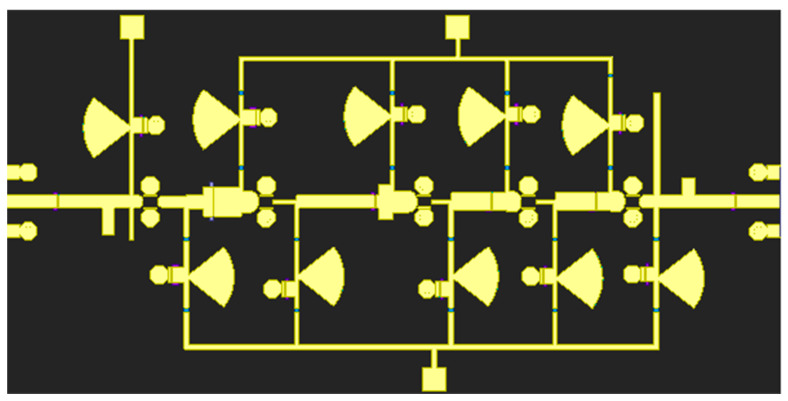
Entire layout of LNA.

The transmission line is composed of the MT1(metal layer), VIA2 (through-hole), and MT2 (metal layer), stacked together in three layers of the same size. The capacitor consists of the MT1 as the lower plate, the MT2 as the upper plate, and the SiN (medium) sandwiched in between. After synthesizing these transmission lines, the design operates at high frequencies in the terahertz band. However, due to the high-frequency nature, the accuracy of the device model is not as precise as in the low-frequency range, and electromagnetic coupling between microstrip lines becomes a significant concern. To address these challenges, it is crucial to perform a reasonable circuit layout after completing the schematic simulation. Directly connecting the RF Microstrips is one strategy aimed at reducing transmission losses. The microstrips are placed relatively far apart, and separate holes are utilized to ground the components requiring grounding. Additionally, in order to enhance design accuracy, it is important to consider the parasitic effects of RF pads and the grounding of transistor sources during the layout phase. Subsequently, a comprehensive layout analysis and full-band electromagnetic field (EM) simulation should be conducted on the circuit, excluding the active devices. Figure 7 illustrates the EM layout model used in the simulation. The overall chip has a square shape with dimensions of 2.6 mm × 1.3 mm. Under bias conditions of $V_{d}$ = 1.8 V and $V_{g}$ = 0 V, the total power consumption is 0.34 W. By adhering to these steps and conducting a comprehensive layout analysis and simulation, this design can achieve improved performance and reduced losses, thereby ensuring the amplifier’s effective operation within the terahertz frequency range.

**Figure 8:**
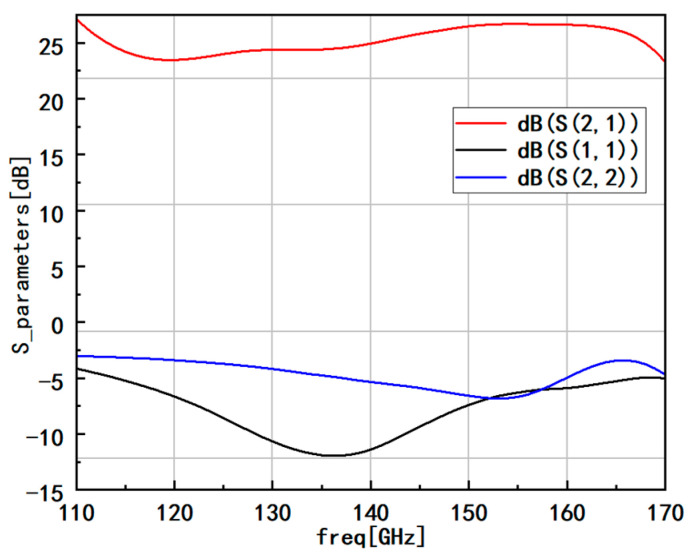
Gain and reflection coefficient of integral amplifier.

Figure 8 illustrates the gain performance of our low-noise amplifier. It is evident that the amplifier’s bandwidth covers the entire frequency band. The gain near the central frequency is approximately 25 dB, with the input reflection coefficient at about −10 dB and the output reflection coefficient at about −5 dB. Across the entire operating frequency range, the gain consistently exceeds 22 dB. This remarkable gain characteristic, coupled with the wide bandwidth coverage and well-maintained reflection coefficients, demonstrates the effectiveness and reliability of the low noise amplifier design. The amplifier’s performance is well suited for various applications within the specified frequency range, ensuring robust signal amplification and transmission capabilities.

**Figure 9:**
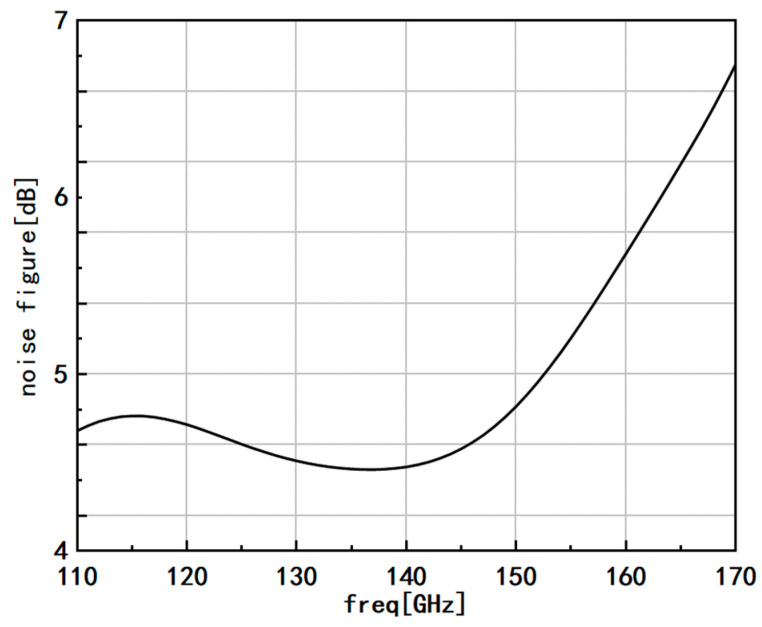
Noise performance of amplifier.

Figure 9 displays the noise performance of the amplifier. The noise figure near the center frequency of 140 GHz is approximately 4.4 dB, and within the frequency band of 110–150 GHz, the noise figure remains below 5 dB. In the frequency range of 150–170 GHz, due to the increasing internal noise of transistors at high frequencies and the additional losses introduced by transmission lines and other components, the noise figure becomes significant. However, it still remains below 7 dB. These noise figures demonstrate the excellent noise characteristics of the low noise amplifier, particularly within the desired frequency range. With noise levels kept low and stable, the amplifier is well equipped to deliver high-fidelity signal amplification, making it suitable for various applications in the terahertz frequency band.

**Figure 10:**
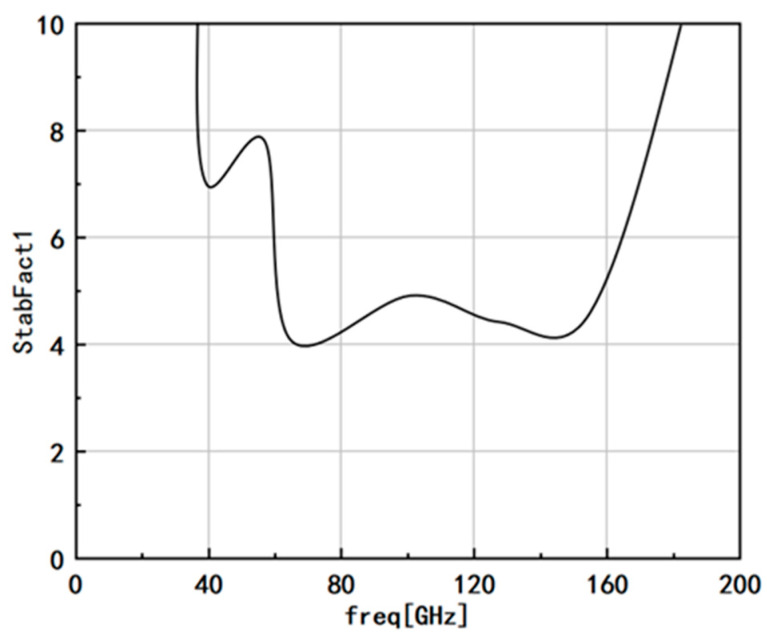
Stability coefficient of amplifier.

Figure 10 illustrates the stability coefficient of the amplifier. To ensure absolute stability, the stability coefficient should be greater than 1. In this study, a stability coefficient exceeding 3.2 was achieved across the full frequency band, indicating excellent stability characteristics of the amplifier. This high-stability coefficient ensures the amplifier’s reliable and consistent performance, making it well suited for applications where stability is critical. The amplifier’s ability to maintain stability across the entire frequency range reinforces its suitability for demanding terahertz communication systems and other high-frequency applications.

**Figure 11:**
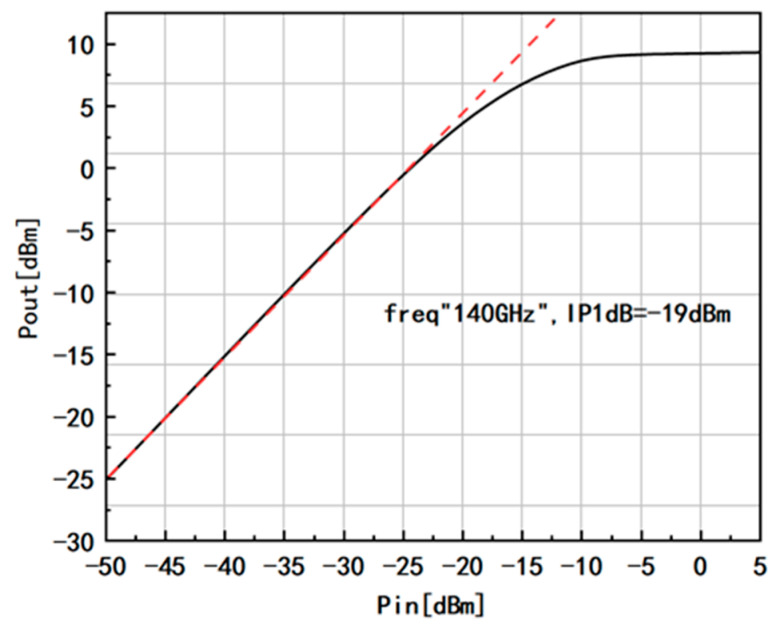
Pout vs. Pin and 1 dB compression point of the LNA.

Figure 11 illustrates the 1 dB compression point output power of the amplifier. At 140 GHz, the output power experiences a 1 dB compression when the input power reaches −19 dBm. This remarkable performance showcases the amplifier’s outstanding linearity and power handling capabilities.

A comparison of the LNA performance determined in this work with that from simulation carried out in previous publications is summarized in Table 2. The superior gain, larger bandwidth, and lower noise of the LNA designed in this paper indicate the success of the optimization strategies employed, showcasing its potential for use in various high-performance terahertz communication systems.

## 5. Conclusions

This article presents the design of a broadband high-gain terahertz-low noise amplifier (LNA) operating in the frequency range of 110–170 GHz. The complete circuit is constructed using InP HMET active devices along with input, output, interstage matching circuits, GSG circuits, and DC bias circuits. The passive components of the circuit are simulated using the momentum tool for electromagnetic analysis, allowing for precise modeling and optimization. The LNA circuit is then subjected to electromagnetic co-simulation, integrating the circuit and electromagnetic simulations. The comprehensive simulation results demonstrate the amplifier’s excellent performance, with a gain of approximately 25 dB, an S11 reflection coefficient below −10 dB, a noise figure of about 4.4 dB, and an input 1 dB compression point of −19 dBm at 140 GHz. The terahertz low-noise amplifier designed in this study exhibits impressive gain and noise figure characteristics, showcasing its potential for applications in communication systems. With its ability to efficiently amplify signals in the terahertz frequency band, this LNA holds promising prospects for various communication and signal processing applications.

## Figures and Tables

**Table 2:** Comparison with existing terahertz low noise amplifiers.

Ref.	Technology	Frequency (GHz)	Gain (dB)	NF (min) (dB)	BW (GHz)
[1]	70 nm InP	110–140	12	6	30
[2]	0.25 μm InP	120–140	13	6	20
[3]	80 nm InP	170–194	7.2	5	24
[10]	80 nm InP	170–200	9.6	-	30
Prop.	70 nm InP	110–170	25	4.4	60

## Data Availability

Data sharing is not applicable.
